# Exploration of the potential association between GLP-1 receptor agonists and suicidal or self-injurious behaviors: a pharmacovigilance study based on the FDA Adverse Event Reporting System database

**DOI:** 10.1186/s12916-024-03274-6

**Published:** 2024-02-14

**Authors:** Jianxing Zhou, You Zheng, Baohua Xu, Songjun Long, Li-e Zhu, Yunhui Liu, Chengliang Li, Yifan Zhang, Maobai Liu, Xuemei Wu

**Affiliations:** 1 Department of Pharmacy, Fujian Medical University Union Hospital, Fuzhou, Fujian China; 2 School of Pharmacy, Fujian Medical University, Fuzhou, Fujian China; 3 School of Medical Imaging, Fujian Medical University, Fuzhou, Fujian China; 4 Department of Respiratory, Shanghai Electric Power Hospital, Shanghai, China; 5 Shanghai Institute of Materia Medica, Chinese Academy of Sciences, Shanghai, China

**Keywords:** GLP-1RAs, Suicidal or self-injurious behaviors, Pharmacovigilance, FAERS database, Type 2 diabetes, Obesity

## Abstract

**Background:**

Establishing whether there is a potential relationship between glucagon-like peptide 1 receptor agonists (GLP-1RAs) and suicidal or self-injurious behaviors (SSIBs) is crucial for public safety. This study investigated the potential association between GLP-1RAs and SSIBs by exploring the FDA Adverse Event Reporting System (FAERS) database.

**Methods:**

A disproportionality analysis was conducted using post-marketing data from the FAERS repository (2018 Q1 to 2022 Q4). SSIB cases associated with GLP-1RAs were identified and analyzed through disproportionality analysis using the information component. The parametric distribution with a goodness-of-fit test was employed to analyze the time-to-onset, and the Ω shrinkage was used to evaluate the potential effect of co-medication on the occurrence of SSIBs.

**Results:**

In total, 204 cases of SSIBs associated with GLP-1RAs, including semaglutide, liraglutide, dulaglutide, exenatide, and albiglutide, were identified in the FAERS database. Time-of-onset analysis revealed no consistent mechanism for the latency of SSIBs in patients receiving GLP-1RAs. The disproportionality analysis did not indicate an association between GLP-1RAs and SSIBs. Co-medication analysis revealed 81 cases with antidepressants, antipsychotics, and benzodiazepines, which may be proxies of mental health comorbidities.

**Conclusions:**

We found no signal of disproportionate reporting of an association between GLP-1RA use and SSIBs. Clinicians need to maintain heightened vigilance on patients premedicated with neuropsychotropic drugs. This contributes to the greater acceptance of GLP-1RAs in patients with type 2 diabetes mellitus or obesity.

**Graphical Abstract:**

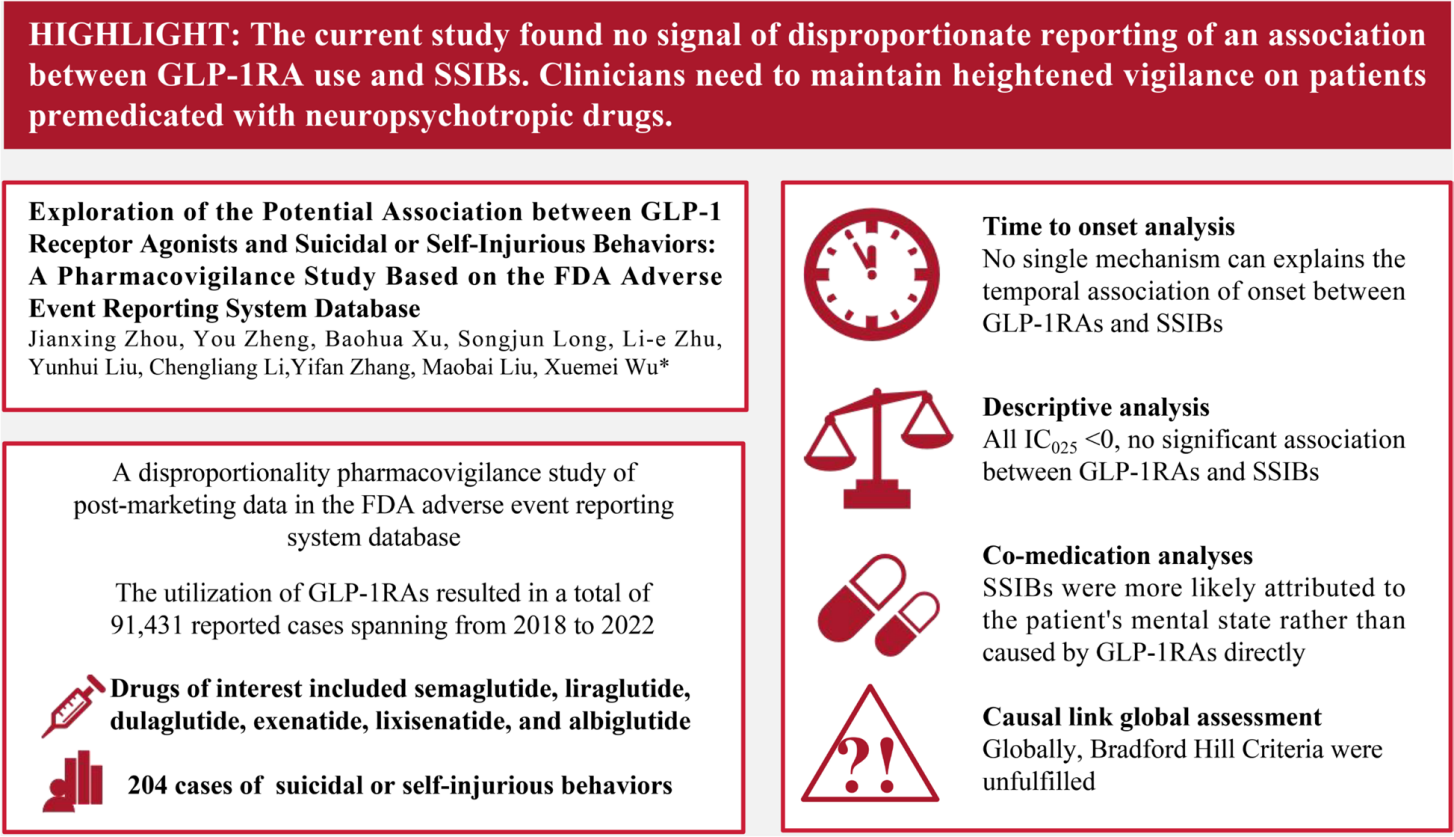

**Supplementary Information:**

The online version contains supplementary material available at 10.1186/s12916-024-03274-6.

## Highlights

• To determine whether there is a potential relationship between glucagon-like peptide 1 receptor agonists (GLP-1RAs) and suicidal or self-injurious behaviors (SSIBs).

• Is there a direct association between GLP-1RAs and SSIBs? No evidence reasonably suggests an association between GLP-1RAs and SSIBs based on clinical characteristics, time-onset, disproportionality, and co-medication analysis.

• Clinicians should pay more attention to the psychiatric status of patients with a history of neuropsychotropic drugs, and more comprehensive monitoring is needed to consider their susceptibility to SSIBs carefully.

**Supplementary Information:**

The online version contains supplementary material available at 10.1186/s12916-024-03274-6.

## Background

Glucagon-like peptide 1 receptor agonists (GLP-1RAs) are modified derivatives of glucagon-like peptide 1 (GLP-1) that simulate the regulatory effect of GLP-1 on blood glucose levels. The primary advantages of GLP-1RAs lie in glucose reduction, cardiorenal benefits, and amelioration of metabolic syndrome, all of which are substantiated by a plethora of evidence-based studies [1–4]. Therefore, GLP-1RAs have emerged as one of the recommended pharmacological interventions for type 2 diabetes mellitus, especially for patients with cardiovascular risk factors and coexisting comorbidities (e.g., chronic kidney disease) [5]. Furthermore, GLP-1RAs exhibit substantial weight reduction effects by facilitating glucose uptake and suppressing appetite [6]. According to the Centers for Disease Control and Prevention, diabetes and obesity rank among the top 10 most economically burdensome chronic diseases in the USA. Given the escalating global prevalence of these diseases, there is an unprecedented opportunity to expand the global market for GLP-1RAs.

Since the approval of exenatide for the treatment of type 2 diabetes by the Food and Drug Administration (FDA) in 2005, research on GLP-1 RAs has become a hot topic and new analogs are constantly being introduced to the market. In December 2014, the FDA approved liraglutide as a weight loss drug. Right after this, semaglutide became available with the indication of diabetes in 2017, and then in 2020, its treatment for obesity was also authorized. Global demand for semaglutide and liraglutide exceeds the present supply, which might be related to their off label uses and misuse [7]. Despite the significant potential and advantages of GLP-1RAs, they are inevitably accompanied by a range of possible adverse drug reactions (ADRs), as is the case with any medication. The most prevalent ADRs associated with GLP-1RA therapy encompass gastrointestinal events such as nausea, vomiting, and diarrhea. However, these non-serious ADRs are tolerable in individuals with diabetes and obesity [8–10]. What raises concerns are some of the recent reports of serious ADRs associated with GLP-1RAs: (i) Pfizer’s disclosure of elevated aminotransferases in users of lotiglipron (a novel oral GLP-1RA) is indicative of hepatocellular injury [11]; (ii) Studies revealed an elevated risk of thyroid cancer associated and cholecystitis with the use of GLP-1RAs [12, 13]; (iii) The Icelandic Medicines Agency has submitted 150 reports of GLP-1RAs-related suicidal or self-injurious behaviors (SSIBs) to the European Medicines Agency (EMA) [14]. Following the aforementioned incidents, the development of lotiglipron was discontinued, and drug regulatory agencies in several countries classified GLP-1RAs as a potential risk factor for thyroid cancer, gallbladder, and biliary diseases. However, no studies have investigated the association between GLP-1RAs and SSIBs. SSIBs, recognized as significant ADRs, have garnered widespread acknowledgment as critical factors that may jeopardize patient safety [15]. Studies have demonstrated a consistent year-on-year escalation in patient suicides associated with medication use, intensifying concerns among the general public and clinical professionals regarding this phenomenon [16–18]. Therefore, in the context of the widespread global utilization of GLP-1RAs, it is imperative to investigate whether a potential relationship exists between GLP-1RAs and SSIBs, which is of paramount importance for public and clinical medication safety.

The FDA Adverse Event Reporting System (FAERS) is a comprehensive database of ADRs specifically designed by the FDA to facilitate postmarketing surveillance of drugs and therapeutic biologics [19]. The database includes all ADRs and medication errors documented by the FDA. It facilitates the identification and quantitative analysis of signals indicating disproportionate reporting of ADRs, thereby aiding in the recognition of associations between specific drugs and particular ADRs [20, 21].

This study aimed to explore the morbidity characteristics of SSIBs associated with GLP-1RAs by mining the FAERS database and measuring the association between GLP-1RAs and SSIBs through metrics such as time of onset, disproportionality analysis, and co-medication analysis. The results of this study will have a positive impact on medication safety for patients with type 2 diabetes or obesity, which offering substantial backing for clinical medication choices.

## Methods

### Data sources and study design

The FAERS, one of the largest publicly accessible databases for ADRs, provides researchers with raw data from the FDA website (https://fis.fda.gov/extensions/FPD-QDE-FAERS/FPD-QDE-FAERS.html). To process the extracted raw data from the FAERS database, we utilized Microsoft SQL Server (version 2019; Microsoft Corporation, Redmond, WA, USA). Considering duplicate or implausible reports can lead to significant errors [22], extensive cleaning, and normalization were conducted to ensure interpretable data (detailed process is provided in Additional file 1: Fig S1[23–25]).

The study year was selected on the basis of drug and data availability. At the commencement of this study, the FDA only published data for the first quarter of 2023. Tirzepatide (marketed in September 2022) was excluded due to insufficient data, and semaglutide was the latest drug of interest to be marketed (December 2017). Therefore, we performed a disproportionality analysis of postmarketing data from the FAERS repository to investigate the potential association of SSIBs with GLP-1RAs. Data were selected for each complete year since the latest drug was marketed from 2018 quarter 1 to 2022 quarter 4. Ethical approval was not required as the study was conducted using de-identified publicly available data.

### Definition of cases and drugs of interest

In the FAERS database, ADRs are classified according to the Medical Dictionary for Regulatory Activities (MedDRA) terminology in terms of signs and symptoms, which are called preferred terms (PTs). The ADRs of interest include suicidal ideation, self-injurious ideation, suicidal behaviors, and self-injurious behaviors (see Additional file 1: Table S1 for a detailed list), which are homogenously attributed to a high-level group term (HLGT) named suicidal and self-injurious behavior by MedDRA (version 26.0). The drugs of interest included FDA-approved GLP-1RAs [26]: semaglutide, liraglutide, dulaglutide, exenatide, lixisenatide, and albiglutide. In this study, venlafaxine was used as a positive control because it is significantly associated with SSIBs [27]. Orlistat and empagliflozin were selected as negative controls based on their similarity in the population indicated for GLP-1RAs, without any reported evidence of self-harm or suicide potential in the specification or related studies. The current study encompassed all cases in which GLP-1RAs were administered and listed as primary suspect drugs, secondary suspect drugs, or concomitant drugs for SSIBs.

### Descriptive analysis

Patient demographics (age, sex, and reporter type) and clinical aspects (latency, outcome, and indication) were documented in reports of SSIBs with semaglutide, liraglutide, dulaglutide, exenatide, lixisenatide, and albiglutide.

### Reported time­to-onset analysis

To describe the latency of SSIBs with GLP-1RAs, we performed a time-to-onset (TTO) analysis by selecting the parametric distribution model that demonstrated the best result in the goodness-of-fit test among the Weibull, log-normal, gamma, and exponential distributions. The parametric distribution is characterized by the scale parameter α and shape parameter β [28]. The scale parameter α represents the magnitude of ADRs occurring at the 63.2% quantile within the distribution function. Depending on the value of the shape parameter β, the upper limit of the 95% confidence interval (CI) for β < 1 indicates an initial increase, followed by a decrease in the hazard rate (early-failure type). Conversely, the lower limit of the 95% CI for β > 1 demonstrates an increasing hazard over time (wear-out failure type), whereas the 95% CI includes 1 for β, suggesting a constant hazard rate throughout the exposure period (random failure type). More details about TTO analysis and classification of β can be found in the Additional file 1, sections 4–5.

### Disproportionality analysis

A sequential approach was adopted to systematically address major confounders:

1. An exploratory disproportionality approach comparing GLP-1RAs with all other drugs reported in the FAERS database, using venlafaxine as the positive control and empagliflozin/orlistat as the negative control. The definition of the disproportionality approach can be found in Additional file 1: section 4 [29].
2. We utilized the Bayesian Information Component (IC) to calculate the lower limit of the 95% CI (IC_025_). This approach is more precise than relying on the report odds ratio, particularly in situations with limited cases [30]. The IC calculation procedure can be found in Additional file 1: section 5.
3. False-negative analysis was conducted by artificially augmenting the reported incidence of GLP-1RA-associated SSIBs by 100%, aiming to ascertain whether the absence of positive findings could be attributed to our exclusion of some cases involving the concurrent use of multiple GLP-1RAs. This meticulous examination effectively substantiates the credibility of the negative results.
4. Given the inherent heterogeneity and potential reporting bias in the FAERS database, we conducted a series of sensitivity analyses. First, we excluded case and non-case reports with gastrointestinal events (all PTs belonged to gastrointestinal disorders in the system organ class) in the dataset. This step aimed to reduce the masking effect of gastrointestinal events to avoid possible competitive bias. Furthermore, the main indications in the included cases were type 2 diabetes mellitus and obesity, which are potential risk factors for SSIBs. Therefore, we narrowed the analysis dataset to subject with type 2 diabetes mellitus or weight loss through the indi_pt field (which represents the indication). Previous studies have shown that restricting the analyzed population can mitigate indication bias [21]. IC_025_ was calculated using the processed dataset to conduct the above sensitivity analyses.
5. Considering a low number of expected cases could lead to an insufficient sensitivity to detect disproportionality of relevant strength [31], we reported the sensitivity of a representative IC_025_ to measure the reliability of negative results. The result was deemed confident when the sensitivity to detect representative IC_025_>0.8. The calculation procedure can be found in Additional file 1: Fig S2.

### Co-medication analysis

A case-by-case analysis was performed by two independent pharmacists to identify the combined medications by searching DRUG and THER files of the included 204 cases. When discrepancies existed, a third pharmacist was introduced to make the final decision. We conducted a co-medication analysis of GLP-1RAs and the top 20 most frequently used drugs in cases of SSIBs associated with GLP-1RAs. Calculation of IC_025_ for these drugs is consistent with the procedure described in section 2.5. We used the Ω shrinkage to measure drug–drug interactions because a previous study showed that it is the most conservative method among multiple algorithms [32]. The detection criterion is the lower limit of the 95% CI of the Ω (Ω_025_) > 0. The calculation process of Ω and the list of drugs were described in Additional file 1: Table S2. When at least one neuropsychiatric drug (such as antipsychotics, antidepressants, and anxiolytics) was recorded in the report, the patient was defined to have a medication history.

### Global assessment of the evidence

The Modified Bradford Hill Criteria was used to assess the potential relationships among various available evidence [33–35]. The biological plausibility can assess whether there is a mechanism to support the potential relationship between ADRs and the studied drug. We also considered the strength of the evidence (the magnitude of observed effects) and the consistency of findings across various studies or sources. Additionally, specificity was utilized to assess whether some events were associated with specific factors, and coherence was to examine the temporal sequence of events that aligned with a potential relationship. Lastly, other similar drugs (empagliflozin and Orlistat) were used as a negative control to support the final conclusion.

### Statistical analysis

The disproportionality threshold of the reporting signal was defined as IC_025_ > 0, and a positive drug-drug interaction was defined as Ω_025_ > 0. R (version 4.3.1; R Foundation for Statistical Computing, Vienna, Austria) was used for plotting, TTO analysis, and calculation of IC and Ω. The magnified percentage used in the false-negative analysis was 100%, determined based on a step-by-step strategy (Additional file 1: Fig S3, and Table S3).

## Results

### Descriptive analysis

From 2018 quarter 1 to 2022 quarter 4 of 2018, 57, 74, 51, 20, and 2 reports of SSIBs associated with semaglutide, liraglutide, dulaglutide, exenatide, and albiglutide, respectively, were identified in the FAERS database. Three patients were excluded because of the concomitant use of multiple GLP-1RAs, and no SSIB reports for lixisenatide were detected. The distribution of the reported years exhibited a generally even pattern, with a noticeable upward trend observed in 2022. The majority (54.90%) of the reports on SSIBs utilizing GLP-1RAs were contributed by healthcare professionals. Despite the small amount of missing or unknown data (2.94%), the proportion of reports in females was significantly higher than that in males (64.22 vs. 32.84%, respectively). Among the reports with available data, half of the cases were observed in adults (18–65 years). Type 2 diabetes (34.80%) and weight loss (14.71%) emerged as the most predominant indications, while others accounted for 3.92%, and unknown or missing constituted 46.57% (Table 1).

**Table 1:** Cases characteristics of SSIBs associated with GLP-1RA in the FAERS database

**Categories**	**Semaglutide *N* (%)**	**Liraglutide *N* (%)**	**Dulaglutide *N* (%)**	**Exenatide *N* (%)**	**Albiglutide *N* (%)**	**Total** ***N* (%)**
**Reports of SSIBs**	57	74	51	20	2	204
**Report year**						
**2018**	2 (3.51)	16 (21.62)	12 (23.53)	4 (20.00)	1 (50.00)	35 (17.16)
**2019**	4 (7.02)	11 (14.86)	7 (13.73)	7 (35.00)	0	29 (14.22)
**2020**	8 (14.04)	19 (25.68)	10 (19.61)	4 (20.00)	1 (50.00)	42 (20.59)
**2021**	16 (28.07)	12 (16.22)	8 (15.69)	2 (10.00)	0	38 (18.63)
**2022**	27 (47.37)	16 (21.62)	14 (27.45)	3 (15.00)	0	60 (29.41)
**Reporter **^**a**^						
**Healthcare professional**	30 (52.63)	53 (71.62)	19 (37.25)	9 (45.00)	1 (50.00)	112 (54.90)
**Nonhealthcare professional**	26 (45.61)	21 (28.38)	31 (60.78)	6 (30.00)	1 (50.00)	85 (41.67)
**Unknown or missing**	1 (1.75)	0	1 (1.96)	5 (25.00)	0	7 (3.43)
**Sex**						
**Male**	18 (31.58)	20 (27.03)	19 (37.25)	9 (45.00)	1 (50.00)	67 (32.84)
**Female**	38 (66.67)	52 (70.27)	29 (56.86)	11 (55.00)	1 (50.00)	131 (64.22)
**Unknown or missing**	1 (1.75)	2 (2.70)	3 (5.88)	0	0	6 (2.94)
**Age category, years**						
**Juvenile (< 18)**	0	0	0	3 (15.00)	0	3 (1.47)
**Adult (18–65)**	28 (49.12)	43 (58.11)	24 (47.06)	7 (35.00)	0	102 (50.00)
**Seniors (> 65)**	1 (1.75)	7 (9.46)	10 (19.61)	2 (10.00)	0	20 (9.80)
**Unknown or missing**	28 (49.12)	24 (32.43)	17 (33.33)	8 (40.00)	2 (100.00)	79 (38.73)
**Median (IQR)**	43 (35–52)	47 (36–57)	55 (46–67)	40 (25–54)	/	/
**Indication**						
**Type 2 diabetes mellitus**	13 (22.81)	14 (18.92)	30 (58.82)	13 (65.00)	1 (50.00)	71 (34.80)
**Weight loss**	15 (26.32)	15 (20.27)	0	0	0	30 (14.71)
**Others**	1 (1.75)	4 (5.41)	0	3 (15.00)	0	8 (3.92)
**Unknown or missing**	28 (49.12)	41 (55.41)	21 (41.18)	4 (20.00)	1 (50.00)	95 (46.57)
**Outcomes**^**b**^						
**Death**	1 (1.75)	17 (22.97)	3 (5.88)	1 (5.00)	0	22 (10.78)
**Life-threatening**	6 (10.53)	6 (8.11)	3 (5.88)	5 (25.00)	0	20 (9.80)
**Hospitalization**	8 (14.04)	20 (27.03)	19 (37.25)	7 (35.00)	0	54 (26.47)
**Disability**	2 (3.51)	1 (1.35)	2 (3.92)	1 (5.00)	0	6 (2.94)
**Other serious illness**	52 (91.23)	55 (74.32)	37 (72.55)	12 (60.00)	2 (100)	158 (77.45)

*IQR* interquartile range, *SSIBs* Suicidal and Self-Injurious Behaviors, *GLP-1RAs* GLP-1 receptor agonists

^a^Healthcare professionals including reporters such as physicians and pharmacists; nonhealthcare professionals including reporters such as consumer and lawyer

^b^Since a case may experience different clinical outcomes during drug therapy, it is reasonable that the sum percentage of the outcome under this item may exceed 100%

### Reported time-to-onset analysis

**Fig. 1:**
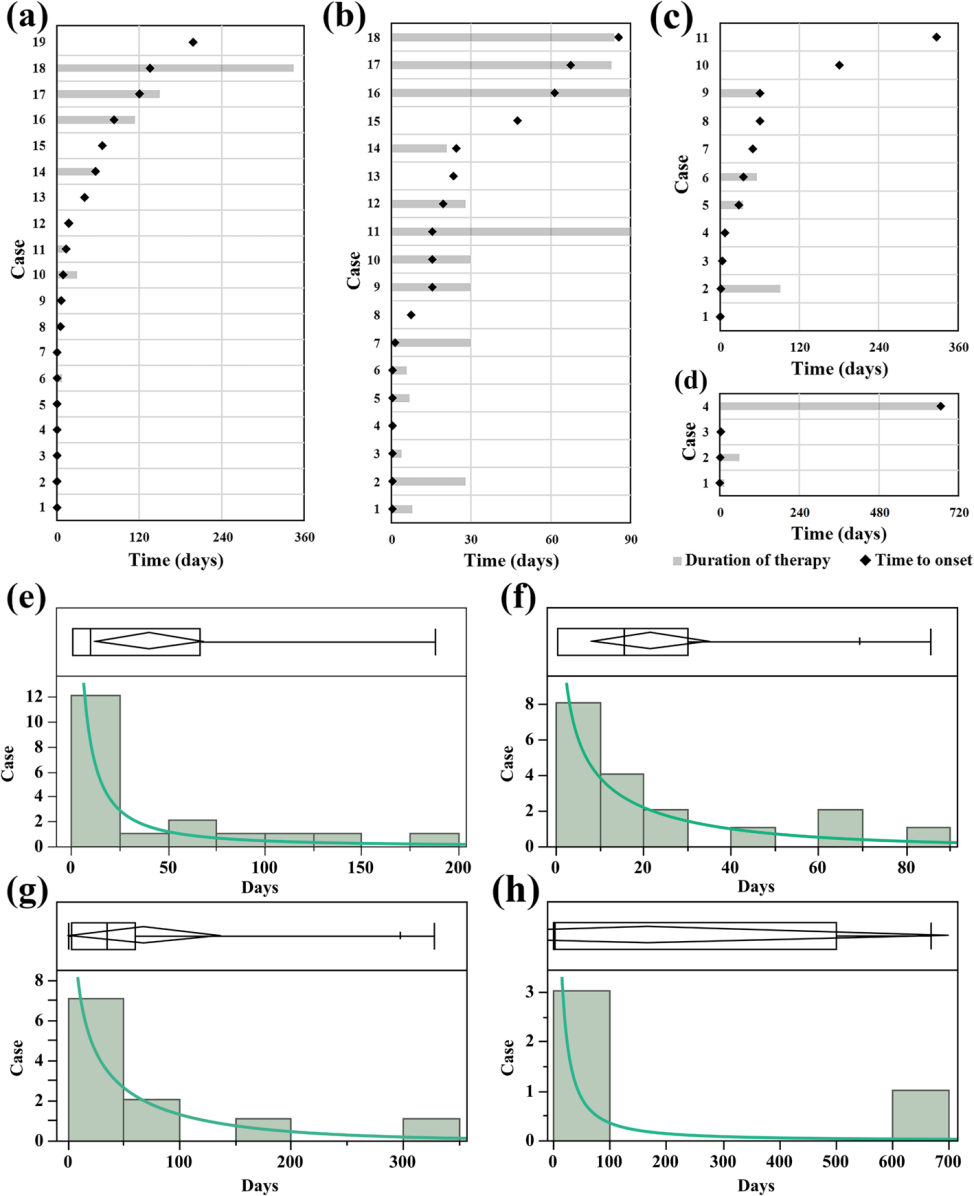
TTO analysis of SSIBs reported for each GLP-1 RA. Reported TTO analysis and duration of treatment of SSIB associated with **a** liraglutide, **b** semaglutide, **c** dulaglutide, and **d** exenatide. Black diamonds represent the TTO of SSIBs following the administration of GLP-1RAs, while gray bars indicate the duration of GLP-1 treatment for each case. Due to limitations in data availability, the duration of therapy could only be plotted for a subset of the cases. Goodness-of-fit test of SSIB associated with **e** liraglutide, **f** semaglutide, **g** dulaglutide, and **h** exenatide. Positioned above the plot is the quantile boxplot. The green bars correspond to the case number of occurrences within the distribution, while the green line represents the fitted curve for the model exhibiting the most optimal outcomes. we adopted log-normal distribution to describe the latency of liraglutide and exenatide, gamma distribution for semaglutide, and Weibull distribution for dulaglutide. The raw data of TTO analysis in the 52 valid cases can be found in Additional file 1: Table S7. TTO, time-to-onset; SSIBs, Suicidal and Self-Injurious Behaviors; GLP-1 RA, GLP-1 receptor agonist

A total valid case of 52 was used for TTO analysis (Fig. 1). Among them, three cases reported SSIBs on 1 day, 1 day, and 3 days after the cessation of GLP-1RAs (1 case for liraglutide and 2 cases for semaglutide), which might be related to the delayed ADR caused by the interested drugs.

In analysis of TTO based on parameter distributions and the valid cases, the median TTOs (Interquartile range) for SSIBs associated with semaglutide, liraglutide, dulaglutide, and exenatide were 15.5 (0.5–24.25, *n* = 18), 9.5 (0.5–61.5, *n* = 19), 35.5 (6.5–60.5, *n* = 11), and 2.5 (1.5–169.5, *n* = 4) days, respectively (albiglutide was not included in the TTO analysis due to insufficient data). The results of goodness-of-fit performance test (Additional file 1: Table S4) indicated that a log-normal model was the best one to describe the latency of liraglutide- and exenatide-related SSIBs. A Gamma model was suitable for semaglutide-related SSIBs, and a Weibull model described the latency of dulaglutide-related SSIBs well. Semaglutide and dulaglutide were classified as early failure types, exenatide as a random failure type, and liraglutide as a wear-out failure type (Additional file 1: Table S5 for raw data).

### Disproportionality analysis

**Fig. 2:**
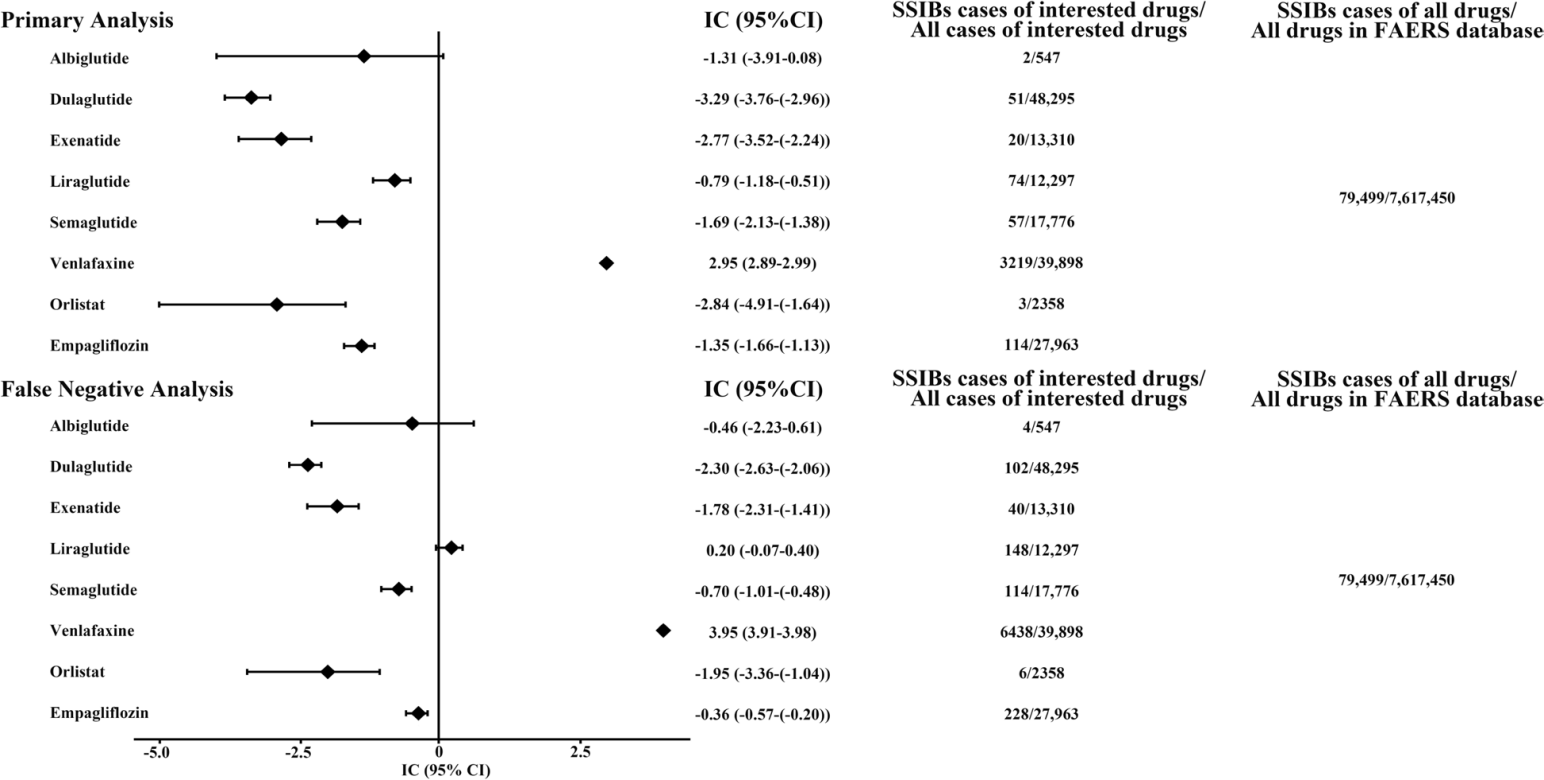
IC_025_ of SSIBs associated with GLP-1 RA in the primary analysis and false negative analysis. When IC_025_ > 0, a disproportionate reporting signal was detected. FAERS, US Food and Drug Administration Adverse Event Reporting System; IC, information component; SSIBs, Suicidal and Self-Injurious Behaviors; GLP-1 RA, GLP-1 receptor agonist

**Fig. 3:**
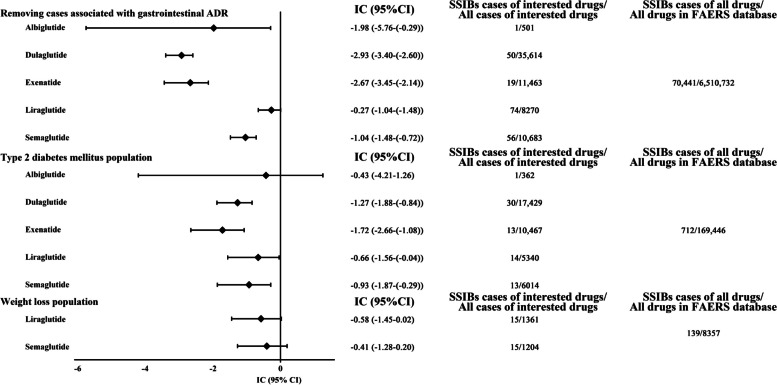
IC_025_ of SSIBs associated with GLP-1 RA in the sensitivity analysis. When IC_025_ >0, a disproportionate reporting signal was detected. FAERS, US Food and Drug Administration Adverse Event Reporting System; IC, information component; SSIBs, Suicidal and Self-Injurious Behaviors; GLP-1 RA, GLP-1 receptor agonist

First, primary analysis was conducted to compare GLP-1RAs with other drugs in the FAERS database. The evaluated GLP-1RAs were semaglutide (IC_025_ = − 2.13), liraglutide (IC_025_ = − 1.18), dulaglutide (IC_025_ = − 3.76), exenatide (IC_025_ = − 3.52), and albiglutide (IC_025_ = − 3.91), none of which exhibited a signal of disproportionate reporting. Negative (empagliflozin, IC_025_ = − 1.66; orlistat, IC_025_ = − 4.91) and positive (venlafaxine, IC_025_ = 2.89) controls confirmed the internal validity of the database. We then performed a false-negative analysis in which the results of the disproportionate analysis of all GLP-1 drugs with SSIBs did not satisfy IC_025_ > 0 after expanding the number of reports by 100%, and the negative and positive control results remained valid (Fig. 2). Finally, IC_025_ < 0 was also observed in the sensitivity analyses (Fig. 3). The sensitivity to detect this IC_025_ was > 0.8 for all GLP-1 RAs in primary and false-negative analyses. In some subgroup analyses, there are some sensitivities to detect this IC_025_ was < 0.8 for albiglutide and exenatide. The raw data for the above analysis can be found in the Additional file 1: Table S8.

### Co-medication analysis

We conducted a co-medication analysis of the top 20 medications with the highest reported frequency of use for all GLP-1RA-associated SSIBs (considering the same reported cases, 23 medications were included). Multiple neuropsychiatric drugs, including bupropion, quetiapine, and aripiprazole, were identified, and disproportionality analysis with SSIBs yielded a reporting signal. Further examination revealed that 81 patients had a history of neuropsychiatric drugs, and 63 patients used more than one neuropsychiatric drug (Additional file 1: Fig S4). Subsequent Ω shrinkage results indicated no potential for drug–drug interactions between all GLP-1RAs and neuropsychiatric drugs (all Ω_025_ < 0; refer to Additional file 1: Table S6 for the list of top 20 medications’ IC_025_, and Ω_025_).

### Causal relationship global assessment

Globally, the Bradford Hill Criteria were not fulfilled, as indicated by the strength of the disproportionality and its consistency throughout the analysis, temporal relationships, and biological plausibility, thus unsupporting a likely causal association between GLP-1RAs (semaglutide, liraglutide, dulaglutide, exenatide, lixisenatide, and albiglutide) and SSIBs (Table 2).

**Table 2:** Global assessment through adapted Bradford Hill Criteria

**Criteria**	**Description**	**Source/method**
**Strength of the association**	Although ICs are not measures of risk, the strength of the disproportionality both in primary (vs. all other drugs) and false negative analysis suggests a negative signal	Disproportionality analysis
**Analogy**	The irrelevance was also demonstrated for other anti-diabetic/anti-obesity drugs (empagliflozin and Orlistat), which were used as a negative control in this study	Disproportionality analysis and labels
**Biological plausibility/empirical evidence**	GLP-1RAs are also proposed to have pro-cognitive effects. Particularly in terms of dual therapeutic mechanisms potentially improving both central nervous system deficits and metabolic burden [36]. There is no evidence to support that GLP-1RA will cause SSIBs	Disproportionality analysis and literature
**Consistency**	Results of disproportionality approaches were consistent in false negative	Disproportionality analysis
**Coherence**	A randomized, controlled trial reports three adolescent cases associated with suicidal ideation/behavior using liraglutide. However, the participant who committed suicide, who was in the liraglutide group, had a history of attention deficit–hyperactivity disorder and there was one suicide attempt in the liraglutide and placebo groups, respectively [37]. An exploratory pooled analysis reported 34/3291 suicidal ideation with liraglutide. But no between-treatment imbalances in suicidal ideation/behavior or depression were noted through prospective questionnaire assessments [38]. A case report describes two instances of depression associated with semaglutide [39].	literature search
**Specificity**	The results of primary and false-negative analysis showed no association between GLP-1RAs and SSIBs. The co-medication analysis indicated that the occurrence of SSIBs was more likely to be related to the patient's own mental state.	Disproportionality and co-medication analysis
**Temporal relationship**	Available data suggested that there were cases of SSIBs after discontinuation of GLP-1RA, but further studies could not be performed due to missing data	Time-to-onset analysis
**Reversibility**	This criterion is of limited value here as there is not enough data on rechallenge and de-challenge in the FAERS database	Descriptive

## Discussion

The current study conducted a comprehensive analysis comprising four key objectives: first, we characterized the clinical features of SSIB cases related to GLP-1RA by analyzing postmarketing data from the FAERS database; second, we developed a latency model for these cases; third, we concluded the lack of disproportional reporting signals regarding GLP-1RAs and SSIBs; finally, we examined co-administration patterns of GLP-1RAs and potential drug–drug interactions with other medications to explore their impact on the reporting frequency of SSIBs.

Initially, we compared the clinical characteristics of patients treated with GLP-1RAs and SSIBs. Reports from 2018 to 2021 exhibited an overall average distribution. However, an uptrend in both ADR with case and non-case was observed in 2022, possibly reflecting the increased prescription of GLP-1RAs in recent years. Among the available data on adverse events, a significantly higher proportion of females (64.22%) than males (32.84%) reported SSIBs. Adults constituted the primary reporting population, accounting for the majority of all reports (juvenile: 1.47%, adults: 50.00%, and the elderly: 9.80%). The aforementioned sex disparities are also evident in epidemiological investigations of suicide attempts and suicidal ideation, which have demonstrated a higher prevalence of self-harming behaviors among females than among males. Notably, this disparity is particularly pronounced in obese individuals [40, 41]. With regard to age, physicians should exert heightened caution when considering the use of such medications in pediatric and geriatric populations, given their safety profiles [42]. Previous studies have also indicated that adults may exhibit a higher susceptibility to suicidal and self-harming tendencies than other age groups [43], potentially contributing to a higher number of reported cases among adults. Furthermore, between 2018 and 2022, 91,431 cases involving GLP-1RAs were reported, with the proportions of females, males, and unknown being 53.77, 38.79, and 7.44%, respectively. The proportion of children, adults, elderly individuals, and those of unknown age were 0.20, 27.02, 19.29, and 53.48%, respectively. We believe that the higher reporting by females and adults may also be related to the distribution of reports. By analyzing these data patterns and trends, we gained preliminary insights into the potential association between GLP-1RAs and SSIBs.

Next, we delved into the potential association between GLP-1RAs and TTO of SSIBs. Our analysis aimed to determine whether these drugs might influence the development of SSIBs by some common mechanism. GLP-1RAs are a class of drugs used to treat type 2 diabetes that can simulate the regulatory effect of GLP-1 on blood glucose levels. Although these GLP-1RAs differ in molecular structure and duration of action, their pharmacological mechanisms are similar [44, 45]. Therefore, we hypothesized that if GLP-1RAs had potential mechanistic associations with SSIBs, their pathogenic patterns would be similar. However, our results revealed unexpected findings. We observed three different pathogenic models for the drugs in the TTO analysis. For example, semaglutide and dulaglutide were categorized as early-failure types, exenatide was determined to be a random failure type, and liraglutide was classified as a wear-out failure type. This suggests that no single mechanism (tolerance effect/accumulation effect) explains the temporal association of onset between GLP-1RAs and SSIBs. Thus, the diversity of the onset patterns observed for GLP-1RAs appears to challenge the above assumptions, making it difficult to conclude a clear relationship between them.

We then performed an exploratory analysis of GLP-1RAs against all other drugs in the FAERS database using the same methodology used in previous pharmacovigilance studies [20, 21]. Negative and positive controls were used to determine the internal validity of the database. Orlistat and empagliflozin served as negative controls, and venlafaxine served as a positive control. The results for these control drugs were consistent with the information available in the literature and labels, confirming the reliability of our study in terms of methodology and data analysis. The results of the disproportionate analysis showed that none of the GLP-1RAs showed signaling with SSIBs. These negative results imply no direct association between GLP-1RAs and SSIBs. Considering that we discarded three cases involving the concurrent use of multiple GLP-1RAs, we further performed a false-negative analysis to avoid false-negative results. However, even with a 100% increase in the number of reports, the results of the disproportionate analysis between all GLP-1RAs and SSIBs did not meet the criterion of IC_025_ > 0. Considering the masking effect of gastrointestinal events and the occurrence of SSIBs caused by underlying diseases, sensitivity analyses were performed. After restricting case and non-case according to the screening conditions, the results remained negative. In addition, further subgroup analyses for different ages and ADR also yielded negative results (Additional file 1: Fig S5–6). The sensitivity to detect this IC_025_ of GLP-1 RAs was > 0.8, suggesting that the absence of a signal is meaningful. However, in some subgroup analyses, the absence of a signal (sensitivity < 0.8) for albiglutide and exenatide is possibly attributed to the reduced number of drugs and ADRs for screening conditions. Validation of the above analysis further confirmed the lack of evidence of an association between GLP-1RAs and SSIBs.

We further analyzed the top 20 concomitant medications with the highest frequency reported in SSIBs associated with GLP-1RAs for co-medication analysis. Multiple neuropsychiatric drugs, including bupropion, quetiapine, and aripiprazole, were included among these medications. Further delving into the use of neuropsychiatric drugs in the cases included in this study, we found that 81 individuals with GLP-1RA-related SSIBs had a history of neuropsychiatric drug use. The results of disproportionality analysis performed for these medications and SSIBs showed a reporting signal, and studies have suggested that individuals who use these medications may be at a higher risk of SSIBs [46]. These results indicate an association between neuropsychiatric drugs and GLP-1RA-related SSIBs. Increased adverse metabolic effects (diabetes, weight gain, dyslipidemia, and increased cardiovascular risk) are common during neuropsychiatric drug treatment [36]. Therefore, the potential co-medication of neuropsychiatric drugs and GLP-1RAs is high. We further explored potential drug interactions between GLP-1RAs and neuropsychiatric drugs by performing an Ω shrinkage analysis. The results showed that none of the GLP-1RAs showed possible drug interactions with neuropsychiatric drugs. In combination with neuropsychiatric drugs, GLP-1RAs did not increase the risk of developing SSIBs. Moreover, GLP-1RAs have cognition-promoting effects and activate the GLP-1R/cAMP/PKA pathway, thus providing a new intervention for the treatment of depression and reduction of SSIBs [36, 47, 48]. These studies provide a comprehensive and accurate biological perspective for a deeper understanding of the association between GLP-1RAs and SSIBs. Our view is beginning to lean toward the idea that the occurrence of SSIBs is more likely to be related to the mental state of the patient rather than GLP-1RAs.

Based on the above results and assessment of available research information, we adopted the adjusted Bradford Hill Criteria from previous publications and assessed the point-by-point link between GLP-1RAs and SSIBs. In general, the current evidence does not fully meet the adjusted Bradford Hill Criteria. Studies have reported liraglutide-associated suicide and semaglutide-associated depression in the coherence criterion [37–39]. However, the researchers concluded that liraglutide was not associated with the occurrence of SSIBs. In the randomized controlled trial (RCT) by Kelly et al. [37], a suicide case in the liraglutide group was reported to have a history of attention deficit hyperactivity disorder, which is a potential factor of SSIBs. Meanwhile, one adolescent attempted suicide in each group (the placebo group and the liraglutide group), highlighting that adolescents are a vulnerable population that should be monitored carefully. In another report of semaglutide-associated depression [39], the individual had a long history of depression, making it challenging to rule out the interference of disease recurrence. Furthermore, in the program of Semaglutide Treatment Effect in People with Obesity [49–52], no SSIBs were reported except for some mild or moderate psychiatric disorders(e.g., insomnia, anxiety) were observed. Through a comprehensive retrospective analysis of the strength of the association, analogy, and consistency metrics of GLP-1RAs and SSIBs, we concluded that there was no evidence reasonably suggesting an association between GLP-1RAs and SSIBs. However, the RCT studies, case reports, and the FAERS database case all highlighted that we should be aware of co-administered neuropsychotropic drugs that may associated with SSIBs. Clinicians should recognize that patients undergoing polypharmacy require heightened vigilance, comprehensive monitoring, and specialized counseling.

Our study has some limitations. First, as a spontaneous reporting system, the FAERS has inherent shortcomings, such as duplicate records with variable information quality. Despite manual corrections and deletions, only a few duplicate cases may exist [53, 54]. Second, due to the limited availability of TTO data, the number of valid cases may be inadequate. Consequently, the conclusions drawn from the TTO analysis should be regarded as low-quality evidence. Third, false-negative analysis is a test that we propose based on the specific situation of this study. However, it is not yet widely used in the field of pharmacovigilance. Therefore, its application warrants careful consideration. Fourth, the SSIBs defined in our study were PTs included for Suicidal and self-injurious behaviors (HLGT level). Given the differences in the MedDRA’s categorization rules, some potential PTs that were not included may exist, e.g., depression suicidal (belonging to HLGT level, depressed mood disorders and disturbances) and intentional overdose (belonging to HLGT level, overdoses, and underdoses NEC).

The FAERS database has become the most widely used ADR database in recent years. By mining and analyzing the FAERS database, this study addressed concerns about the potential association between GLP-1RAs and SSIBs and helped dispel public concerns regarding drug safety. The majority of SSIB cases reported in the current RCT and post-marketing study are not considered to be related to GLP-1 RAs. The disproportionate analyses conducted concurrently with our study, based on the FAERS database with varying year spans, also indicate no direct association between GLP-1RAs and SSIBs [55, 56]. In a retrospective analysis utilizing electronic health records, researchers noted that semaglutide can even reduce the risk of suicide compared to non-GLP-1 RAs anti-obesity medications [57]. However, given the rarity of the occurrence of SSIBs, the size of the RCT, and the limitations of the retrospective studies, higher-quality and larger prospective trials are needed to determine the credibility of the current conclusions.

## Conclusions

This study explored the relationship between GLP-1RAs and SSIBs through disproportionality analysis. This study found no evidence reasonably suggesting an association between GLP-1RA and SSIBs based on clinical characteristics, TTO, disproportionality, and co-medication analysis. Comparatively, clinicians should pay more attention to the psychiatric status of patients with a history of neuropsychotropic drugs, and more comprehensive monitoring is needed to consider their susceptibility to SSIBs carefully. The results of this study have a positive impact on medication safety for patients with type 2 diabetes or obesity, which is important in maintaining public health and providing strong support for clinical medication decisions.

### Supplementary Information

**Additional file 1:** **Fig S1.** The main steps in the processing of the FAERS database. **Fig S2.** Summary of the workflow for calculation of the sensitivity. **Fig S3.** Changes in IC025 values for each GLP-1 RA after gradual expansion of case number. **Fig S4.** Information on 81 cases with a history of psychotic drug use. **Fig S5.** Comparison of IC025 for Each GLP-1 RA in Different Age Groups of Adults. **Fig S6.** Comparison of IC025 for suicide or self-injury. **Table S1.** List of adverse reactions of SSIBs included in this study. **Table S2.** The 4 × 2 contingency table for signal detection of drug-drug interaction. **Table S3.** Cases of monotherapy and polytherapy with GLP-1 RAs. **Table S4.** The performance test of goodness-of-fit among four parametric distribution models. **Table S5.** Time-to-onset analysis of SSIBs associated with GLP-1 RA in the FAERS database. **Table S6.** IC025 and Ω025 of top 23 medications. **Table S7.** The raw data of time to onset analysis in the 52 valid cases. Table S8. The raw data from primary, false-negative, sensitivity and subgroup analyses in calculate disproportionality.

## Data Availability

The datasets generated and/or analyzed during the current study are available in the US FAERS database (https://fis.fda.gov/extensions/FPD-QDE-FAERS/FPD-QDE-FAERS.html). The code generated and/or analyzed in the current study is available from the corresponding author on reasonable request.

## Abbreviations

GLP-1RAs: Glucagon-like peptide 1 receptor agonists
GLP-1: Glucagon-like peptide 1
ADRs: Adverse drug reactions
SSIBs: Suicidal or self-injurious behaviors
EMA: European Medicines Agency
FDA: The Food and Drug Administration
FAERS: The Food and Drug Administration Adverse Event Reporting System
MedDRA: The Medical Dictionary for Regulatory Activities
TTO: Time-to-onset
CI: Confidence interval
IC: Information Component
