# Supplementary material for: Exploration of the potential association between GLP-1 receptor agonists and suicidal or self-injurious behaviors: a pharmacovigilance study based on the FDA Adverse Event Reporting System database
Source: BMC Med. 2024 Feb 14;22:65. doi: 10.1186/s12916-024-03274-6 (PMC10865629; doi:10.1186/s12916-024-03274-6)
Supplement: Supplementary file 1 — Additional file 1: Fig S1. The main steps in the processing of the FAERS database. Fig S2. Summary of the workflow for calculation of the sensitivity. Fig S3. Changes in IC025 values for each GLP-1 RA after gradual expansion of case number. Fig S4. Information on 81 cases with a history of psychotic drug use. Fig S5. Comparison of IC025 for Each GLP-1 RA in Different Age Groups of Adults. Fig S6. Comparison of IC025 for suicide or self-injury. Table S1. List of adverse reactions of SSIBs included in this study. Table S2. The 4 × 2 contingency table for signal detection of drug-drug interaction. Table S3. Cases of monotherapy and polytherapy with GLP-1 RAs. Table S4. The performance test of goodness-of-fit among four parametric distribution models. Table S5. Time-to-onset analysis of SSIBs associated with GLP-1 RA in the FAERS database. Table S6. IC025 and Ω025 of top 23 medications. Table S7. The raw data of time to onset analysis in the 52 valid cases. Table S8. The raw data from primary, false-negative, sensitivity and subgroup analyses in calculate disproportionality. [file 12916_2024_3274_MOESM1_ESM.docx]

**Additional file**

**Title:**

**Exploration of the Potential Association between GLP-1 Receptor Agonists and Suicidal or Self-Injurious Behaviors: A Pharmacovigilance Study Based on the FDA Adverse Event Reporting System Database**

Jianxing Zhou, MS^1,2^, You Zheng, MS^1,2^, Baohua Xu, MS^1,2^, Songjun Long, BS^3^, Li-e Zhu, MS^1^, Yunhui Liu, MS^1^, Chengliang Li, BS^4^ Yifan Zhang, PhD^5^, Maobai Liu, MS^1^, Xuemei Wu, PhD^1,2*^

^1^ Department of Pharmacy, Fujian Medical University Union Hospital, Fuzhou, Fujian, China

^2^ School of Pharmacy, Fujian Medical University, Fuzhou, Fujian, China

^3^ School of Medical Imaging, Fujian Medical University, Fuzhou, Fujian, China

^4^ Department of Respiratory, Shanghai Electric Power Hospital, Shanghai, China

^5^ Shanghai Institute of Materia Medica, Chinese Academy of Sciences, Shanghai, China

^*^ Corresponding author: Xuemei Wu. Ph.D.

Email: wuxuemei@fjmu.edu.cn

**1. The main steps in the processing of the FAERS database**

Microsoft SQL Server (version 2019; Microsoft Corporation., Redmond, WA, United States) was used to extract the required original data in the FAERS database. We processed ADR reports from the first quarter of 2018 to the fourth quarter of 2022 and obtained 28,693,780 ADR records from 8,857,300 cases. Considering that duplicated or incredible reports may produce serious errors, extensive cleaning and normalization were conducted first.

Drug and ADR names were standardized through the Orange Book, PubChem, Drug Bank, and Medical Dictionary for Regulatory Activities (MedDRA). According to MedDRA, ADRs were coded into preferred terms (PTs), and the corresponding system organ class (SOC), high-level group term (HLGT), and high-level term (HLT) were listed to make it possible to compare the potential signal strength on the same level. Considering the credibility of the results, we processed duplicate data by the following criteria: when caseid and fda_dt were identical, duplicate records under the same case were removed by keeping the latest data of fda_dt. In addition, we carried out a double-check of overlapping key field reports. Specifically, when two rows of data contained the same primaryid, caseid, event_dt, drugname, active substances, pt, sex, and age, we thoroughly checked the remaining fields to ensured that no duplicate reports were included in the final dataset. We removed data with quality defects, such as age >120 years, same event code but different gender, and conflicting medication information. The detailed processing steps of the FAERS database was displayed in Fig S1.

**
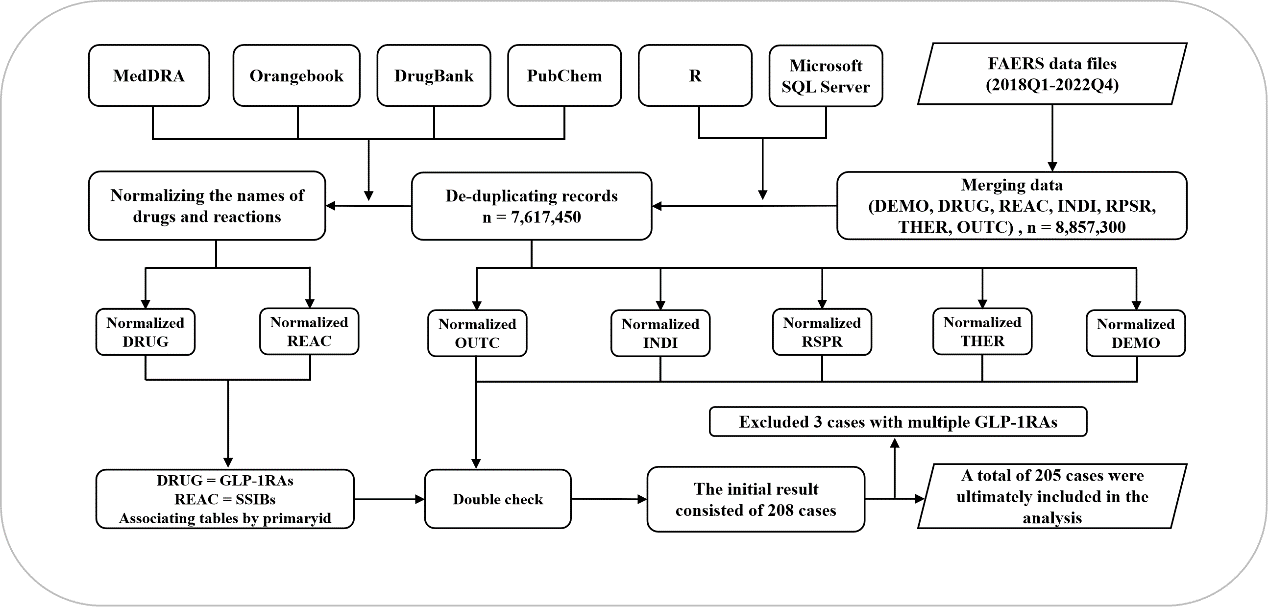
**

**Fig S1.** The main steps in the processing of the FAERS database. DEMO: demographics; DRUG: drug; REAC: reaction; INDI: indication; RPSR: reporting source; THER: therapy; OUTC: outcome. All seven tables above are from the FAERS data file. SSIBs, Suicidal and Self-Injurious Behaviors; GLP-1 RAs, GLP-1 Receptor Agonists.

**2. Definition of suicidal and self-injurious behaviors**

**Table S1.** List of adverse reactions of SSIBs included in this study

| High-level term | Preferred terms | Lowest level term |
| --- | --- | --- |
| Suicidal and self-injurious behavior | **Assisted suicide** | **Assisted suicide, Death with dignity, Physician assisted suicide** |
| Suicidal and self-injurious behavior | **Completed suicide** | **Accomplished suicide, Completed suicide, Suicide, Suicide (accomplished)** |
| Suicidal and self-injurious behavior | **Intentional self-injury** | **Deliberate self-harm, Deliberate self-injury, Intentional self-injury, Parasuicide, Repeated parasuicide, Self inflicted laceration, Self injurious behavior, Self injurious behavior without suicidal intent,** **Self injurious behaviour, Self injurious behaviour without suicidal intent, Self mutilation** |
| Suicidal and self-injurious behavior | **Self-injurious ideation** | **Self-injurious ideation, Thoughts of self harm** |
| Suicidal and self-injurious behavior | **Suicidal behavior** | **Preparatory actions toward imminent suicidal behavior, Preparatory actions toward imminent suicidal behaviour, Suicidal behavior, Suicidal behaviour, Suicide gesture** |
| Suicidal and self-injurious behavior | **Suicidal ideation** | **Active suicidal ideation, Death wishes, Life weariness, Passive suicidal ideation, Suicidal ideation, Suicidal intention, Suicidal plans, Suicidal tendency** |
| Suicidal and self-injurious behavior | **Suicide attempt** | **Attempted suicide, Autolytic attempt, Suicide attempt, Suicide attempt by drug overdose, Suicide attempt other than overdose, Unsuccessful suicide** |
| Suicidal and self-injurious behavior | **Suicide threat** | **Suicide threat** |
| Suicidal and self-injurious behavior | **Suspected suicide** | **Suspected suicide** |
| Suicidal and self-injurious behavior | **Suspected suicide attempt** | **Suspected suicide attempt** |

**3. Calculation of time-to-onset**

$$\begin{aligned} TTO=D_{1}-D_{2}+0.5\#\left( 1 \right) \end{aligned}$$

$$\begin{aligned} DOT=D_{3}-D_{2}+0.5\#\left( 2 \right) \end{aligned}$$

D1: onset date of ADR, from FAERS database's event_dt field

D2: administration start date, from FAERS database's start_dt field

D3: administration end date, from FAERS database's end_dt field

TTO: time-to-onset

DOT: duration of treatment

**4. Definition of the disproportionality approach**

When medicinal products are marketed, case reports of suspected adverse drug reactions (ADRs) are reported to spontaneous reporting systems on a national level. One task is to detect and investigate possible new side-effects of these drugs. All case reports are filed in databases at the National Centres as well as sent onto the WHO Collaborating Centre for International Drug Monitoring (the Uppsala Monitoring Centre). Usually, trained assessors regularly examine every incoming reported combination between a drug and a suspected ADR for possible signals in a case by case analysis. A systematic continuous review of the combinations present in the database is necessary to optimize the primary goal of spontaneous reporting systems, i.e. monitoring for unexpected or unknown ADRs or signal detection. The WHO defines a signal as: ‘Reported information on a possible causal relationship between an adverse event and a drug, of which the relationship is unknown or incompletely documented previously’. Often, a limited number of reports represent a signal. Because of increasingly large numbers of case reports being stored in databases, adequate signal detection without automated quantitative screening is becoming time-consuming and inefficient, because of the sheer load of information to be assessed.

In quantitative signal detection, combinations of a drug and a clinical event that are disproportionately highly represented in the database may represent an important signal based upon a difference from the background frequency. Subsequently, these combinations must still be analyzed and interpreted by the critical human mind. In contrast to hypothesis testing where quantitative estimates are used to express the frequency of a signal, in spontaneous reporting systems, they are used to determine the probability of a combination being a signal or not, based on disproportionate reporting.

The use of a measure of disproportionality is currently applied in various national spontaneous reporting centers as well as in the Uppsala Monitoring Centre. Several point estimates like the Reporting Odds Ratio (ROR), Proportional ADR Reporting Ratio (PRR) or Yule’s Q have been used, in combination with additional estimators of the precision of point estimates such as the Chi-square test or the lower limits of the 95% confidence intervals of the point estimates. Furthermore, the chance of the number of reports being reported on a certain combination, under the assumption that no relationship exists between the reported suspected ADR and the suspected medication, can be calculated by means of the Poisson probability. Another approach is the use of Bayesian logic, specifying the relation between the prior and posterior probability before and after linking data fields, and adding new data to the database, currently being used, for example, by the Uppsala Monitoring Centre in the Bayesian Confidence Propagation Neural Network analysis (BCPNN). This relationship is expressed as the ‘information component’ (IC).

**5. Calculatio definition of the disproportionality approach Bayesian information component**

$$\begin{aligned} IC=\log_{2}\left( \frac{N_{\mathrm{observed}}+ 0.5}{N_{\mathrm{expected}}+ 0.5} \right)\#\left( 3 \right) \end{aligned}$$

$$\begin{aligned} N_{\mathrm{expected}}=\frac{\left( N_{\mathrm{drug}}*N_{\mathrm{effect}} \right)}{N_{\mathrm{total}}}\#\left( 4 \right) \end{aligned}$$

$$\begin{aligned} \mathrm{IC}_{025}=\log_{2}\left( \frac{N_{\mathrm{observed}}+ 0.5}{N_{\mathrm{expected}}+ 0.5} \right)-3.3*\left( N_{\mathrm{observed}}+0.5 \right)^{-\frac{1}{2}}-2*\left( N_{\mathrm{observed}}+0.5 \right)^{-\frac{3}{2}}\#\left( 5 \right) \end{aligned}$$

$$\begin{aligned} \mathrm{IC}_{975}=\log_{2}\left( \frac{N_{\mathrm{observed}}+ 0.5}{N_{\mathrm{expected}}+ 0.5} \right)+2.4*\left( N_{\mathrm{observed}}+0.5 \right)^{-\frac{1}{2}}-0.5*\left( N_{\mathrm{observed}}+0.5 \right)^{-\frac{3}{2}}\#\left( 6 \right) \end{aligned}$$

N_expected_: the number of case reports expected for the drug-ADR pairs.

N_observed_: the actual number of case reports for the drug-ADR pairs.

N_effect_: the number of case reports for the ADR, regardless of the drug.

N_total_: the total number of case reports in the database.

N_drug_: the number of case reports for the drug, regardless of the ADR.

**6. Calculation of the sensitivity**


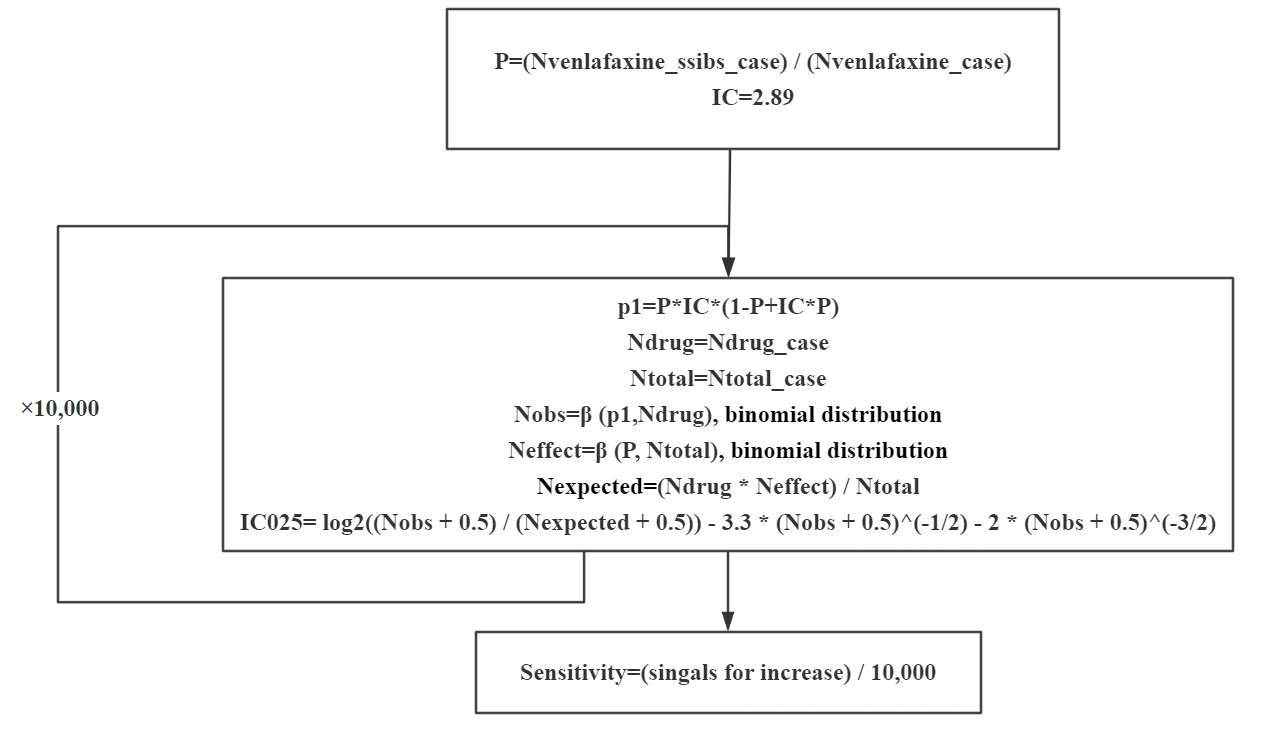


**Fig S2**. Summary of the workflow for calculation of the sensitivity.

P, SSIBs frequency in the FAERS database.

N_venlafaxine_ssibs_case_, the number of case reports for SSIBs, regardless of the drug.

N_venlafaxine_case_, the number of case reports for venlafaxine, regardless of the ADR.

N_total_case_, the number of cases in the FAERS database.

IC=2.89, to assess sensitivity, an IC_025_ of 2.89 was chosen, because venlafaxine associated with SSIBs by this IC_025_≥0 in comparison to GLP-1 RAs.

p_1_, the case frequency in the group of interest.

N_exp_: the number of case reports expected for the drug-SSIBs pairs.

N_obs_: the actual number of case reports for the drug-SSIBs pairs.

N_effect_: the number of case reports for SSIBs, regardless of the drug.

N_total_: the total number of case reports in the database.

N_drug_: the number of case reports for the drug, regardless of the ADR.

**7. Calculation of drug-drug interaction signal (Ω)**

**Table S2.** The 4 × 2 contingency table for signal detection of drug-drug interaction.

|  | Target AE | Other AEs | Total |
| --- | --- | --- | --- |
| Concomitant use of drug D1 and drug D2 | n111 | n110 | n11+ |
| drug D1 without drug D2 | n101 | n100 | n10+ |
| drug D2 without drug D1 | n011 | n010 | n01+ |
| Neither drug D1 nor drug D2 | n001 | n000 | n00+ |
| Total | n++1 | n++0 | n+++ |

*n*: the number of reports.

$$\Omega={log}_{2}\frac{n_{111}+0.5}{E_{111}+0.5} \ldots\left( 7 \right)$$

$$f_{00}=\frac{n_{001}}{n_{00+}}, f_{10}=\frac{n_{101}}{n_{10+}}, f_{01}=\frac{n_{011}}{n_{01+}}, f_{11}=\frac{n_{111}}{n_{11+}} \ldots\left( 8 \right)$$

Where, *n* is the number of reports shown in the 4 × 2 contingency table.

$$g_{11}=1-\frac{1}{\max\left( \frac{f_{00}}{1-f_{00}}, \frac{f_{10}}{1-f_{10}} \right)+ \max\left( \frac{f_{00}}{1-f_{00}}, \frac{f_{01}}{1-f_{01}} \right)- \frac{f_{00}}{1-f_{00}} +1} \ldots\left( 9 \right)$$

When *f*_10_ < *f*_00_ (which denote no risk of ADR caused by *drug D*_1_), the most sensible estimator *g*_11_ = max (*f*_00_, *f*_01_) is yielded and the *vice versa* when *f*_01_ < *f*_00_.

$$E_{111}=g_{11}\times n_{11+} \ldots(10)$$

$$\mathrm{Var}\left( \Omega_{0} \right)=\mathrm{Var}\left( {log}_{2}\frac{n_{111}}{E_{111}} \right)\approx\frac{1}{n_{111}{\log\left( 2 \right)}^{2}} \ldots(11)$$

Where, *n*_111_ is the number of reports and *E*_111_ is the expected value.

$$\Omega_{025}=\Omega-\frac{\phi\left( 0.975 \right)}{ln(2)\sqrt{n_{111}}} \ldots(12)$$

Where, *ϕ* (0.975) is 97.5% of the standard normal distribution.

**8. False-negative analysis**

Monotherapy with GLP-1 RAs was used in 204 cases, while polytherapy was used only in the other 3 cases (namely, with combinations of liraglutide+semaglutide, exenatide+semaglutide, and liraglutide+dulaglutide, respectively). If we treat polytherapy cases as multiple cases of monotherapy, the number of potentially discarded cases was as follows: 2 cases of semaglutide, 2 cases of liraglutide, 1 case of dulaglutide, and 1 case of exenatide. As shown in Table S1, the maximum discarded percent derived from the calculation based on the frequency of occurrence was 5.0%. To test whether the negative result was an artifact due to the limited cases, we artificially increased the cases of monotherapy with a stepwise expansion at 10% intervals. Fig S1 shows that no drug showed a positive signal until the expansion reached 110%. In the final analysis, we defined 100% as the upper limit of the expansion. This value was significantly higher than 5%, which can effectively account for the confidence of negative results.

**Table S3.** Cases of monotherapy and polytherapy with GLP-1 RAs

| GLP-1 RA | **Semaglutide** | **Liraglutide** | Dulaglutide | **Exenatide** | **Albiglutide** |
| --- | --- | --- | --- | --- | --- |
| Monotherapy (n) | **57** | **74** | **51** | **20** | **2** |
| Polytherapy (n) | **2** | **2** | **1** | **1** | **0** |
| Discarded percent (%) | **3.51** | **2.70** | **1.96** | **5.00** | **0** |


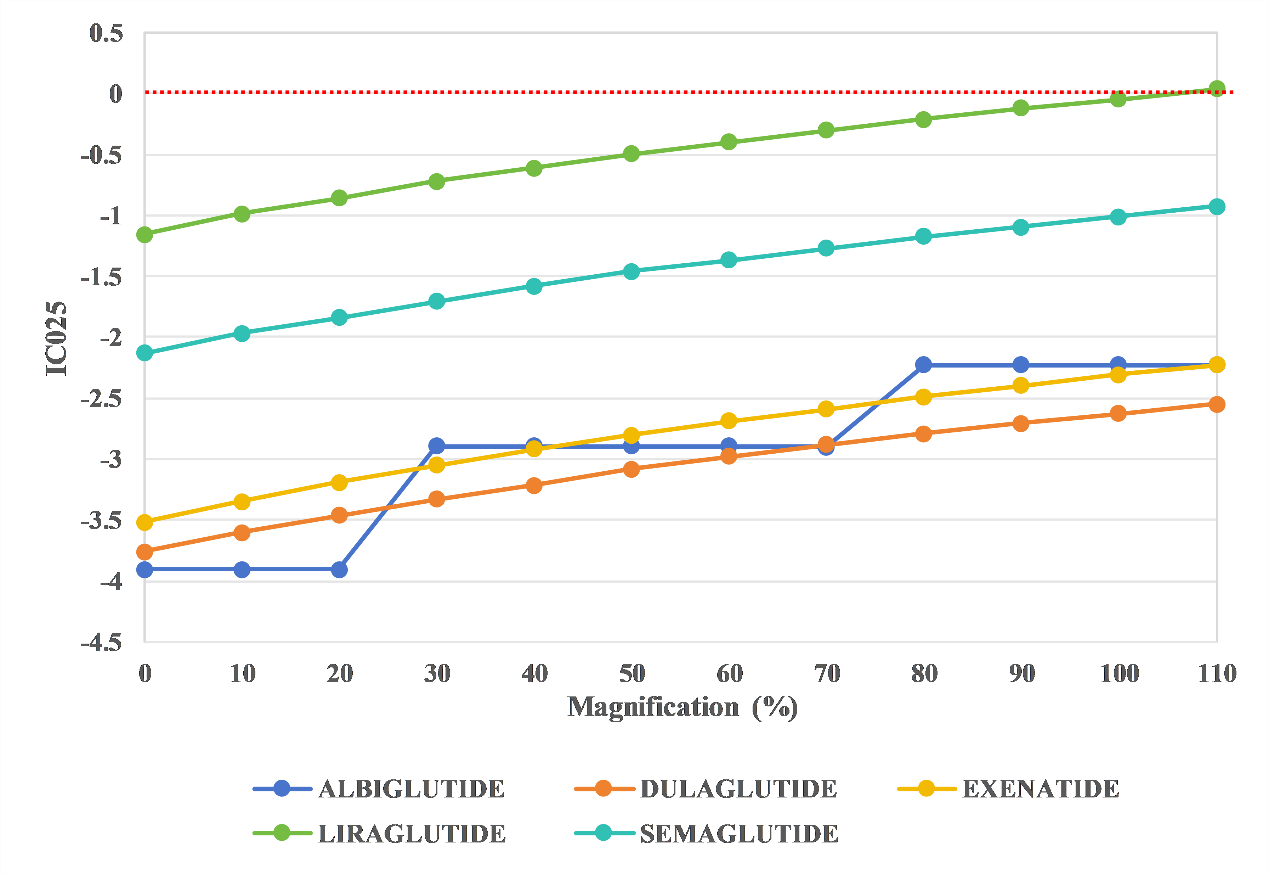


**Fig S3.** Changes in IC_025_ values for each GLP-1 RA after gradual expansion of case number. The red dashed line indicates IC_025_ = 0, and a significant signal was defined as IC_025_ > 0. Rounding to the nearest is applied to determine the value when there are decimals.

**9. The goodness-of-fit performance test results**

**Table S4.** The performance test of goodness-of-fit among four parametric distribution models

| Model name | AICc | BIC | -2*Log L(β) | Selection evidence |
| --- | --- | --- | --- | --- |
| For SSIBs with semaglutide | | | | |
| Weibull | 144.02 | 145.00 | 139.22 | Gamma model presented the smallest AICc and -2*Log L(β), showed good fit |
| Log-normal | 145.12 | 146.10 | 140.31 |  |
| Gamma | 143.60 | 144.58 | 138.80 |  |
| Exponent | 148.79 | 149.43 | 146.54 |  |
| For SSIBs with liraglutide | | | | |
| Weibull | 165.40 | 166.53 | 160.65 | Log-normal model presented the smallest AICc and -2*Log L(β), showed good fit |
| Log-normal | 165.20 | 166.34 | 160.45 |  |
| Gamma | 165.51 | 166.65 | 160.76 |  |
| Exponent | 180.29 | 181.00 | 178.05 |  |
| For SSIBs with exenatide | | | | |
| Weibull | 51.66 | 38.43 | 35.66 | Log-normal model presented the smallest AICc and -2*Log L(β), showed good fit |
| Log-normal | 50.41 | 37.19 | 34.42 |  |
| Gamma | 52.48 | 39.25 | 36.48 |  |
| Exponent | 52.99 | 50.38 | 48.99 |  |
| For SSIBs with dulaglutide | | | | |
| Weibull | 116.15 | 115.44 | 110.65 | Weibull model presented the smallest AICc and -2*Log L(β), showed good fit |
| Log-normal | 117.10 | 116.39 | 111.60 |  |
| Gamma | 116.23 | 115.52 | 110.72 |  |
| Exponent | 117.49 | 117.45 | 115.05 |  |

SSIBs, Suicidal and Self-Injurious Behaviors; AICc, BIC and -2*Log L(β) are indicator of goodness of fit in JMP Pro® version 16 (SAS Institute, Cary, NC, USA). More details about their meaning please refer to Likelihood, AICc, and BIC (https://community.jmp.com/t5/Statistical-Thinking-for/Variable-Selection-in-Generalized-Regression/ta-p/272012)

**10. The parametric distribution of the good fit model**

In the analysis of goodness-of-fit among four parametric distribution models for time-event data, where a shape parameter (β) not equal to 1 indicates that the risk is not constant, i.e., the hazard (or risk) of an event varies over time from the time calculated from the start of treatment to the occurrence of the event. A statistical test whose null hypothesis is that the shape parameter is equal to 1 exists and can be used to test whether there is a potential relationship between treatment initiation and event. After fitting a model to the time-event data, if the shape parameter is not equal to one, the hazard function is not constant, and the event's occurrence is temporally related to the date of first exposure. Based on this, the researchers defined β<1 as early failure type, which means that adverse events are likely to occur in the early stages of treatment. Moreover, when the 95% CI of β includes 1, researchers consider it a random failure type, meaning that adverse events are unrelated to the date of first exposure. When β > 1, researchers consider it to be wear-out type, which means that adverse events are more likely to occur after a long period of medication before they are more likely to occur.

**Table S5.** Time-to-onset analysis of SSIBs associated with GLP-1 RA in the FAERS database

| Categories | Semaglutide | Liraglutide | Dulaglutide | Exenatide |
| --- | --- | --- | --- | --- |
| Reports of SSIBs | 57 | 75 | 51 | 20 |
| Case with valid data for analysis | 18 | 19 | 11 | 4 |
| Median days | 15.5 | 9.5 | 35.5 | 2.5 |
| Scale parameter α (95% CI) | 43.04 (20.81-120.51) | 1.99 (0.92-3.06) | 49.82 (16.32-141.65) | 1.87 (-1.64-5.38) |
| Shape parameter β (95% CI) | 0.50 (0.28-0.83) | 2.26 (1.69-3.22) | 0.64 (0.38-0.97) | 2.76 (0.85-4.68) |

α, scale parameter, represents the scale of the distribution function as the quantile in which 63.2% of AEs occur. β, shape parameter, could be used to confirm the distribution type: early failure type (β<1), random failure type (95% CI of β include 1), and wear-out type (β>1). It should be noted the report counts of semaglutide, liraglutide, dulaglutide and exenatide were not equal to counts in Table 1 due to the limitations in data availability.

95% CI, 95% confidence interval; Aes, adverse events; FAERS, US Food and Drug Administration Adverse Event Reporting System; SSIBs, Suicidal and Self-Injurious Behaviours.

**11. Drug–Drug interaction**

**Table S6.** IC_025_ and Ω_025_ of top 23 medications

| Drug 1 | Drug 2 | N of SSIBs with drug1+drug2/ N of all ADRs with drug1+drug2 | IC (IC_025_-IC_975_) | Ω (Ω_025_-Ω_975_) |
| --- | --- | --- | --- | --- |
| GLP-1RA | Metformin | 47/17902 | 0.68 (0.62-0.73) | -2.79 (-3.21-(-2.38)) |
| GLP-1RA | Fluoxetine | 21/467 | 3.05 (2.99-3.09) | -0.93 (-1.55-(-0.32)) |
| GLP-1RA | Alprazolam | 16/564 | 2.78 (2.74-2.81) | -1.32 (-2.03-(-0.61)) |
| GLP-1RA | Lisinopril | 15/2609 | 0.63 (0.55-0.69) | -1.48 (-2.21-(-0.75)) |
| GLP-1RA | Insulin aspart | 15/2831 | -0.53 (-0.74-(-0.38)) | -0.97 (-1.70-(-0.24)) |
| GLP-1RA | Gabapentin | 15/1928 | 1.47 (1.42-1.51) | -1.87 (-2.60-(-1.14)) |
| GLP-1RA | Quetiapine | 15/246 | 3.52 (3.49-3.55) | -0.96 (-1.69-(-0.23)) |
| GLP-1RA | Insulin glargine | 14/7080 | -1.62 (-1.81-(-1.49)) | -2.38 (-3.14-(-1.63)) |
| GLP-1RA | Furosemide | 14/1931 | -1.12 (-1.25-(-1.03)) | -0.54 (-1.30-(-0.22)) |
| GLP-1RA | Cyclobenzaprine | 14/342 | 2.34 (2.24-2.41) | -0.36 (-1.12-(-0.40)) |
| GLP-1RA | Insulin degludec | 12/2168 | -0.63 (-1.00-(-0.37)) | -0.90 (-1.72-(-0.09)) |
| GLP-1RA | Escitalopram | 12/470 | 2.39 (2.33-2.44) | -1.08 (-1.90-(-0.26)) |
| GLP-1RA | Bupropion | 11/239 | 3.79 (3.73-3.83) | -1.62 (-2.47-(-0.77)) |
| GLP-1RA | Clonazepam | 11/396 | 2.74 (2.69-2.77) | -1.29 (-2.14-(-0.44)) |
| GLP-1RA | Aripiprazole | 11/241 | 3.18 (3.13-3.22) | -1.03 (-1.88-(-0.17)) |
| GLP-1RA | Trazodone | 10/520 | 2.49 (2.43-2.54) | -1.58 (-2.47-(-0.68)) |
| GLP-1RA | Sodium oxybate | 10/308 | 2.38 (2.28-2.45) | -0.72 (-1.62-(-0.17)) |
| GLP-1RA | Atorvastatin | 10/2987 | -0.24 (-0.33-(-0.16)) | -1.61 (-2.50-(-0.72)) |
| GLP-1RA | Aspirin | 10/3632 | -0.96 (-1.05-(-0.90)) | -1.91 (-2.80-(-1.01)) |
| GLP-1RA | Lansoprazole | 9/588 | -0.73 (-0.91-(-0.59)) | -0.48 (-0.46-(-1.42)) |
| GLP-1RA | Potassium chloride | 7/633 | -0.73 (-0.91-(-0.59)) | -0.06 (-1.01-(-1.13)) |
| GLP-1RA | Fluconazole | 7/752 | -1.11(-1.39-(-0.90)) | -0.17 (-1.24-(-0.90)) |
| GLP-1RA | Clopidogrel | 7/979 | -1.06 (-1.24-(-0.94)) | -0.53 (-1.60-(-0.53)) |

When Ω_025_ > 0, a significant drug–drug interaction signal was detected; When IC_025_ > 0, a significant signal was detected between drug2 and SSIBs; SSIBs, Suicidal and Self-Injurious Behaviours.


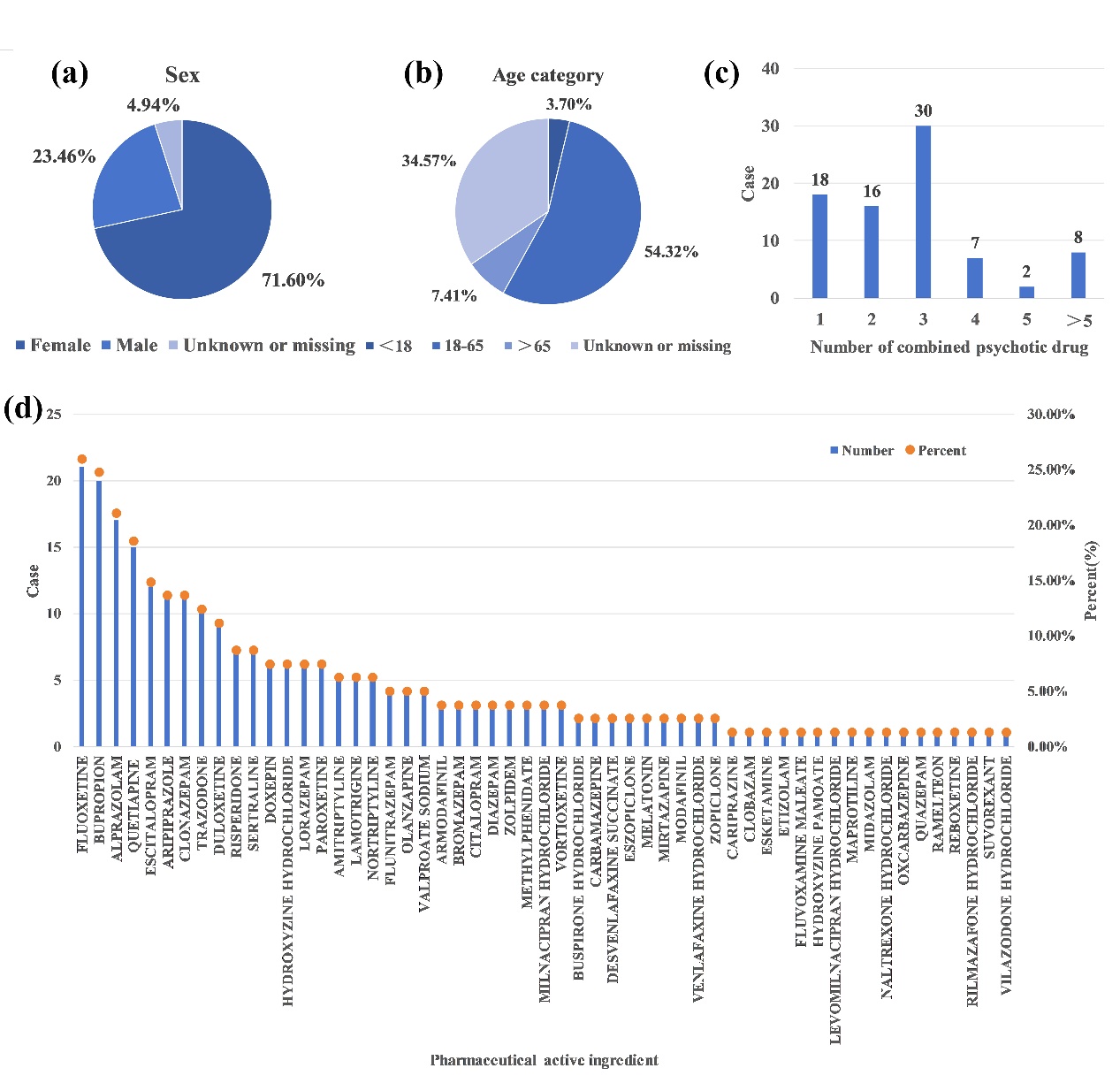


**Fig S4.** Information on 81 cases with a history of psychotic drug use. (a) Sex distribution. (b) Age distribution. (c)Number of combined psychotic drug. (d) Psychotic drug involved and their distribution.

**12. Subgroup analysis**

**
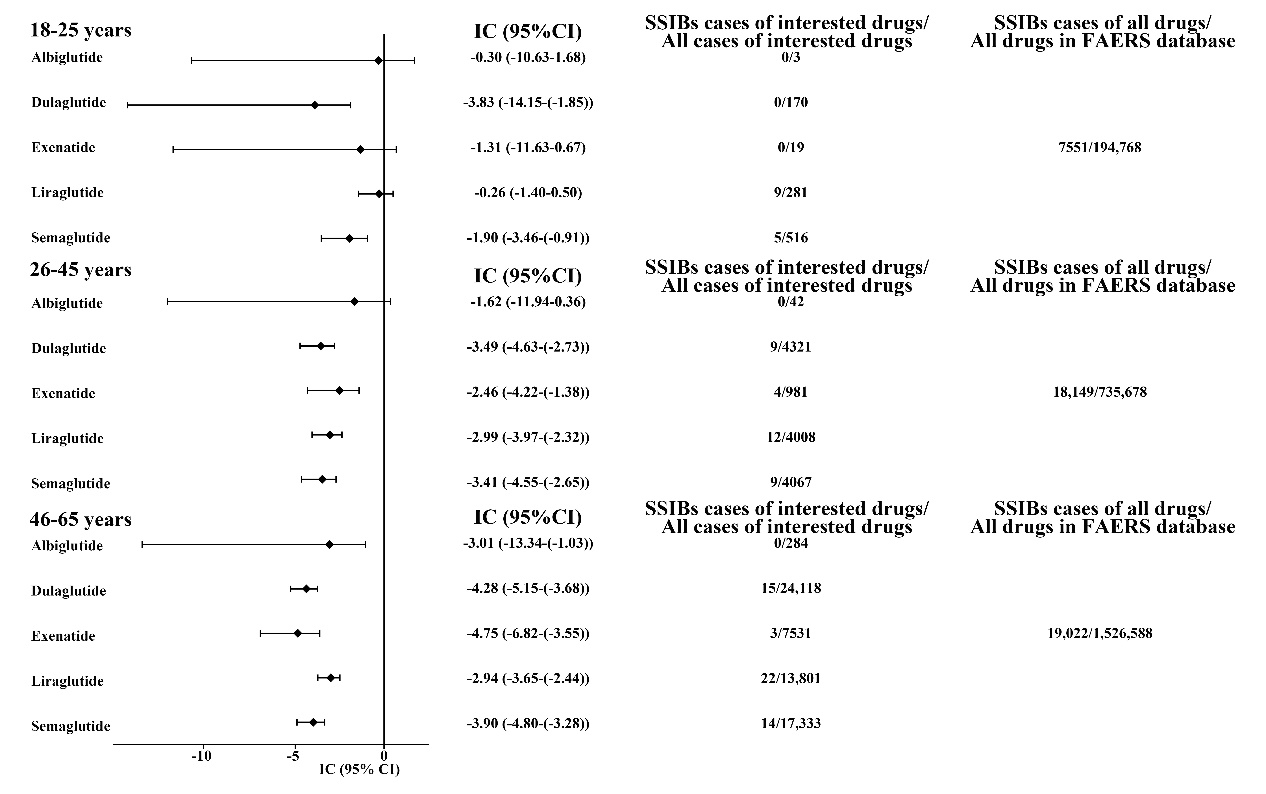
**

**Fig S5.** Comparison of IC_025_ for Each GLP-1 RA in Different Age Groups of Adults

**
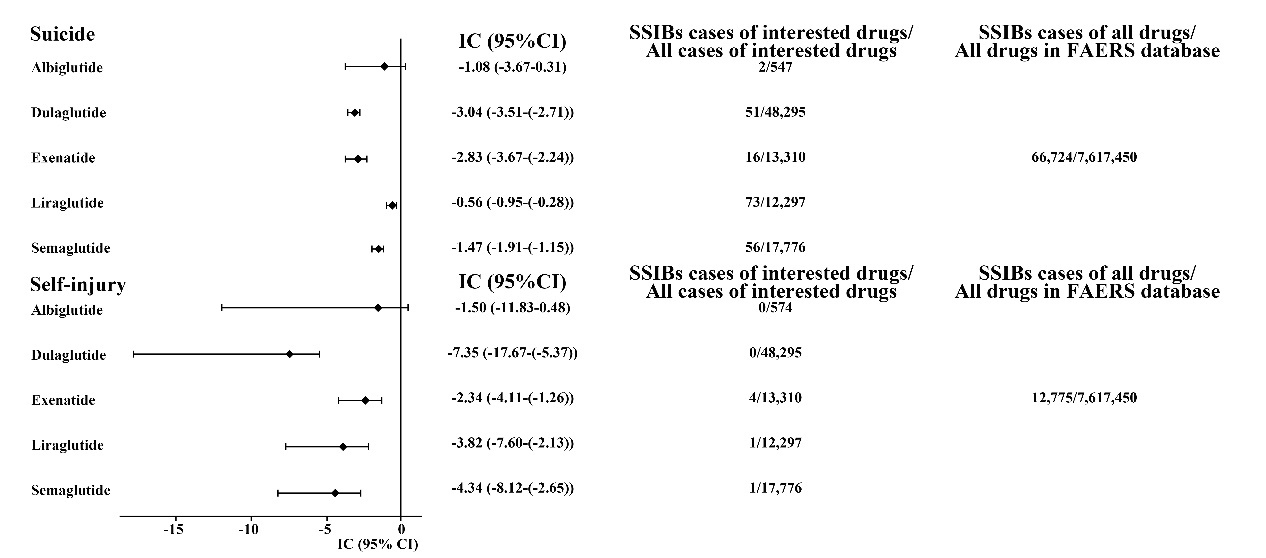
**

**Fig S6.** Comparison of IC_025_ for suicide or self-injury. Self-injury included intentional self-injury and self-injurious ideation. Suicide included suicidal ideation, completed suicide, suicide attempt, suspected suicide, suicidal behavior, suicide threat, and suspected suicide attempt.

**13. Raw data of time to onset analysis**

**Table S7.** The raw data of time to onset analysis in the 52 valid cases

| Case | Duration of  therapy (Days) | Time to onset  (Days) | Case | Duration of  therapy (Days) | Time to onset  (Days) |
| --- | --- | --- | --- | --- | --- |
| semaglutide 1 | 8.5 | 0.5 | liraglutide 9* | 5.5 | 6.5 |
| semaglutide 2 | 28.5 | 0.5 | liraglutide 10 | 30.5 | 9.5 |
| semaglutide 3 | 4.5 | 0.5 | liraglutide 11 | 13.5 | 13.5 |
| semaglutide 4 | 1.5 | 0.5 | liraglutide 12 | / | 17.5 |
| semaglutide 5 | 7.5 | 0.5 | liraglutide 13 | / | 40.5 |
| semaglutide 6 | 6.5 | 0.5 | liraglutide 14 | 58.5 | 56.5 |
| semaglutide 7 | 30.5 | 1.5 | liraglutide 15 | / | 66.5 |
| semaglutide 8 | / | 7.5 | liraglutide 16 | 114.5 | 83.5 |
| semaglutide 9 | 30.5 | 15.5 | liraglutide 17 | 150.5 | 120.5 |
| semaglutide 10 | 30.5 | 15.5 | liraglutide 18 | 345.5 | 135.5 |
| semaglutide 11 | 210.5 | 15.5 | liraglutide 19 | / | 198.5 |
| semaglutide 12 | 28.5 | 19.5 | dulaglutide 1 | / | 0.5 |
| semaglutide 13 | / | 23.5 | dulaglutide 2 | 92.5 | 1.5 |
| semaglutide 14* | 21.5 | 24.5 | dulaglutide 3 | / | 3.5 |
| semaglutide 15 | / | 47.5 | dulaglutide 4 | / | 7.5 |
| semaglutide 16 | 457.5 | 61.5 | dulaglutide 5 | 35.5 | 28.5 |
| semaglutide 17 | 83.5 | 67.5 | dulaglutide 6 | 56.5 | 35.5 |
| semaglutide 18* | 84.5 | 85.5 | dulaglutide 7 | / | 49.5 |
| liraglutide 1 | 1.5 | 0.5 | dulaglutide 8 | / | 60.5 |
| liraglutide 2 | 1.5 | 0.5 | dulaglutide 9 | 60.5 | 60.5 |
| liraglutide 3 | / | 0.5 | dulaglutide 10 | / | 180.5 |
| liraglutide 4 | 1.5 | 0.5 | dulaglutide 11 | / | 327.5 |
| liraglutide 5 | 1.5 | 0.5 | exenatide 1 | 15.5 | 0.5 |
| liraglutide 6 | 8.5 | 0.5 | exenatide 2 | 60.5 | 1.5 |
| liraglutide 7 | 1.5 | 0.5 | exenatide 3 | / | 3.5 |
| liraglutide 8 | 5.5 | 5.5 | exenatide 4 | 666.5 | 666.5 |

*, it means the time to onset is beyond the duration of therapy.

/, it means the duration of therapy is unknowing or missing.

**14. All crude numbers to calculate disproportionality**

**Table S8.** The raw data from primary, false-negative, sensitivity and subgroup analyses in calculate disproportionality

| Analysis | Drugname | N_obs_ | N_drug_ | N_effect_ | N_total_ | N_exp_ | IC_025_ | Sensitivity to detect IC_025_=2.89 |
| --- | --- | --- | --- | --- | --- | --- | --- | --- |
| Primary Analysis | |  |  |  |  |  |  |  |
|  | ALBIGLUTIDE | 2 | 547 | 79499 | 7617450 | 5.71 | -3.91 | 0.91 |
|  | DULAGLUTIDE | 51 | 48295 | 79499 | 7617450 | 504.03 | -3.76 | 1.00 |
|  | EXENATIDE | 20 | 13310 | 79499 | 7617450 | 138.91 | -3.52 | 1.00 |
|  | LIRAGLUTIDE | 74 | 12297 | 79499 | 7617450 | 128.34 | -1.18 | 1.00 |
|  | SEMAGLUTIDE | 57 | 17776 | 79499 | 7617450 | 185.52 | -2.13 | 1.00 |
|  | VENLAFAXINE | 3219 | 39898 | 79499 | 7617450 | 416.39 | 2.89 | 1.00 |
|  | EMPAGLIFLOZIN | 114 | 27963 | 79499 | 7617450 | 291.83 | -1.66 | 1.00 |
|  | ORLISTAT | 3 | 2358 | 79499 | 7617450 | 24.61 | -4.91 | 1.00 |
| False-Negative Analysis | |  |  |  |  |  |  |  |
|  | ALBIGLUTIDE | 4 | 547 | 79499 | 7617450 | 5.71 | -2.23 | 0.91 |
|  | DULAGLUTIDE | 102 | 48295 | 79499 | 7617450 | 504.03 | -2.63 | 1.00 |
|  | EXENATIDE | 40 | 13310 | 79499 | 7617450 | 138.91 | -2.31 | 1.00 |
|  | LIRAGLUTIDE | 148 | 12297 | 79499 | 7617450 | 128.34 | -0.07 | 1.00 |
|  | SEMAGLUTIDE | 114 | 17776 | 79499 | 7617450 | 185.52 | -1.01 | 1.00 |
|  | VENLAFAXINE | 6438 | 39898 | 79499 | 7617450 | 416.39 | 3.91 | 1.00 |
|  | EMPAGLIFLOZIN | 228 | 27963 | 79499 | 7617450 | 291.83 | -0.57 | 1.00 |
|  | ORLISTAT | 6 | 2358 | 79499 | 7617450 | 24.61 | -3.36 | 1.00 |
| Removing Cases Associated with Gastrointestinal ADR | | | |  |  |  |  |  |
|  | ALBIGLUTIDE | 1 | 501 | 70441 | 6510732 | 5.42 | -5.76 | 0.876 |
|  | DULAGLUTIDE | 50 | 35614 | 70441 | 6510732 | 385.32 | -3.40 | 1.00 |
|  | EXENATIDE | 19 | 11463 | 70441 | 6510732 | 124.02 | -3.45 | 1.00 |
|  | LIRAGLUTIDE | 74 | 8270 | 70441 | 6510732 | 89.47 | -0.66 | 1.00 |
|  | SEMAGLUTIDE | 56 | 10683 | 70441 | 6510732 | 115.58 | -1.48 | 1.00 |
| Type 2 diabetes mellitus population | | |  |  |  |  |  |  |
|  | ALBIGLUTIDE | 1 | 362 | 712 | 169446 | 1.52 | -4.21 | 0.286 |
|  | DULAGLUTIDE | 30 | 17429 | 712 | 169446 | 73.24 | -1.88 | 1.00 |
|  | EXENATIDE | 13 | 10467 | 712 | 169446 | 43.98 | -2.66 | 1.00 |
|  | LIRAGLUTIDE | 14 | 5340 | 712 | 169446 | 22.44 | -1.56 | 1.00 |
|  | SEMAGLUTIDE | 13 | 6014 | 712 | 169446 | 25.27 | -1.87 | 1.00 |
| Weight loss population | | |  |  |  |  |  |  |
|  | LIRAGLUTIDE | 15 | 1361 | 139 | 8357 | 22.64 | -1.45 | 0.99 |
|  | SEMAGLUTIDE | 15 | 1204 | 139 | 8357 | 20.03 | -1.28 | 0.99 |
| 18-25 years old in adults | |  |  |  |  |  |  |  |
|  | ALBIGLUTIDE | 0 | 3 | 7551 | 194768 | 0.12 | -10.63 | 0.01 |
|  | DULAGLUTIDE | 0 | 170 | 7551 | 194768 | 6.59 | -14.15 | 0.96 |
|  | EXENATIDE | 0 | 19 | 7551 | 194768 | 0.74 | -11.63 | 0.19 |
|  | LIRAGLUTIDE | 9 | 281 | 7551 | 194768 | 10.89 | -1.40 | 0.99 |
|  | SEMAGLUTIDE | 5 | 516 | 7551 | 194768 | 20.00 | -3.46 | 1.00 |
| 26-45 years old in adults | |  |  |  |  |  |  |  |
|  | ALBIGLUTIDE | 0 | 42 | 18149 | 735678 | 1.04 | -11.94 | 0.20 |
|  | DULAGLUTIDE | 9 | 4321 | 18149 | 735678 | 106.60 | -4.63 | 1.00 |
|  | EXENATIDE | 4 | 981 | 18149 | 735678 | 24.20 | -4.22 | 1.00 |
|  | LIRAGLUTIDE | 12 | 4008 | 18149 | 735678 | 98.88 | -3.97 | 1.00 |
|  | SEMAGLUTIDE | 9 | 4067 | 18149 | 735678 | 100.33 | -4.55 | 1.00 |
| 46-65 years old in adults | | |  |  |  |  |  |  |
|  | ALBIGLUTIDE | 0 | 284 | 19022 | 1526588 | 3.54 | -13.34 | 0.72 |
|  | DULAGLUTIDE | 15 | 24118 | 19022 | 1526588 | 300.52 | -5.15 | 1.00 |
|  | EXENATIDE | 3 | 7531 | 19022 | 1526588 | 93.84 | -6.82 | 1.00 |
|  | LIRAGLUTIDE | 22 | 13801 | 19022 | 1526588 | 171.97 | -3.65 | 1.00 |
|  | SEMAGLUTIDE | 14 | 17333 | 19022 | 1526588 | 215.98 | -4.80 | 1.00 |
| Suicide |  |  |  |  |  |  |  |  |
|  | ALBIGLUTIDE | 2 | 547 | 66724 | 7617450 | 4.79 | -3.67 | 0.83 |
|  | DULAGLUTIDE | 51 | 48295 | 66724 | 7617450 | 423.03 | -3.51 | 1.00 |
|  | EXENATIDE | 16 | 13310 | 66724 | 7617450 | 116.59 | -3.67 | 1.00 |
|  | LIRAGLUTIDE | 73 | 12297 | 66724 | 7617450 | 107.71 | -0.95 | 1.00 |
|  | SEMAGLUTIDE | 56 | 17776 | 66724 | 7617450 | 155.71 | -1.91 | 1.00 |
| Self-injury |  |  |  |  |  |  |  |  |
|  | ALBIGLUTIDE | 0 | 547 | 12775 | 7617450 | 0.92 | -11.83 | 0.13 |
|  | DULAGLUTIDE | 0 | 48295 | 12775 | 7617450 | 80.99 | -17.67 | 1.00 |
|  | EXENATIDE | 4 | 13310 | 12775 | 7617450 | 22.32 | -4.11 | 0.99 |
|  | LIRAGLUTIDE | 1 | 12297 | 12775 | 7617450 | 20.62 | -7.60 | 0.99 |
|  | SEMAGLUTIDE | 1 | 17776 | 12775 | 7617450 | 29.81 | -8.12 | 1.00 |

N_exp_: the number of case reports expected for the drug-ADR pairs.

N_obs_: the actual number of case reports for the drug-ADR pairs.

N_effect_: the number of case reports for the ADR, regardless of the drug.

N_total_: the total number of case reports in the database.

N_drug_: the number of case reports for the drug, regardless of the ADR.
